# Empirical Frequency Bound Derivation Reveals Prominent Mid-Frontal Alpha Associated with Neurosensory Dysfunction in Fragile X Syndrome

**DOI:** 10.21203/rs.3.rs-2855646/v1

**Published:** 2023-04-28

**Authors:** Ernest V Pedapati, John A. Sweeney, Lauren M. Schmitt, Lauren E. Ethridge, Makoto Miyakoshi, Rui Liu, Elizabeth Smith, Rebecca C. Shaffer, Steve W. Wu, Donald L. Gilbert, Paul S. Horn, Craig Erickson

**Affiliations:** Cincinnati Children’s Hospital Medical Center; University of Cincinnati College of Medicine; Cincinnati Children’s Hospital Medical Center; University of Oklahoma Health Sciences Center; Cincinnati Children’s Hospital Medical Center; Cincinnati Children’s Hospital Medical Center; Cincinnati Children’s Hospital Medical Center; Cincinnati Children’s Hospital Medical Center; Cincinnati Children’s Hospital Medical Center; Cincinnati Children’s Hospital Medical Center; Cincinnati Children’s Hospital Medical Center; Cincinnati Children’s Hospital Medical Center

**Keywords:** Fragile X Syndrome, Translational medicine, Thalamocortical, Electroencephalography, Gamma Oscillations, Neurodevelopmental Disorders

## Abstract

The FMR1 gene is inactive in Fragile X syndrome (FXS), resulting in low levels of FMRP and consequent neurochemical, synaptic, and local circuit neurophysiological alterations in the fmr1 KO mouse. In FXS patients, electrophysiological studies have demonstrated a marked reduction in global alpha activity and regional increases in gamma oscillations associated with intellectual disability and sensory hypersensitivity. Since alpha activity is associated with a thalamocortical function with widely distributed modulatory effects on neocortical excitability, insight into alpha physiology may provide insight into systems-level disease mechanisms. Herein, we took a data-driven approach to clarify the temporal and spatial properties of alpha and theta activity in participants with FXS. High-resolution resting-state EEG data were collected from participants affected by FXS (n = 65) and matched controls (n = 70). We used a multivariate technique to empirically classify neural oscillatory bands based on their coherent spatiotemporal patterns. Participants with FXS demonstrated: 1) redistribution of lower-frequency boundaries indicating a “slower” dominant alpha rhythm, 2) an anteriorization of alpha frequency activity, and 3) a correlation of increased individualized alpha power measurements with auditory neurosensory dysfunction. These findings suggest an important role for alterations in thalamocortical physiology for the well-established neocortical hyper-excitability in FXS and, thus, a role for neural systems level disruption to cortical hyperexcitability that has been studied primarily at the local circuit level in animal models.

## Introduction

The FMR1 gene is inactive in Fragile X syndrome (FXS), which results in reduced Fragile X Mental Retardation Protein (FMRP) production and varying levels of intellectual disability, autistic characteristics, and sensory hypersensitivity (Baker et al. 2019; Hagerman et al. 2017). Despite a considerable understanding of molecular and microcircuit alterations in the *Fmr1*^−/−^ KO (Dahlhaus 2018), systems-level changes in the human brain and their relationship to neurosensory function and behavior remain poorly understood in FXS. EEG studies have become increasingly used in the FXS field, both in patients and mouse models, to provide insight into central disorder mechanisms at the level of the brain systems (Ewen et al. 2019; Zhang et al. 2018). We and others have identified reproducible, group-level abnormalities in human resting-state EEG and auditory evoked potentials in FXS associated with intellectual disability, neuropsychiatric symptoms, and sensory hypersensitivity (Ethridge et al. 2019; Ethridge et al. 2016; Wang et al. 2017b). These changes include 1) a global reduction in alpha power (8–12 Hz) and an increase in theta power (3.5–7.5 Hz) and 2) an increase in background gamma power (> 30 Hz) (Ethridge et al. 2019; Ethridge et al. 2016; Musumeci et al. 1988; Van der Molen and Van der Molen 2013; Wang et al. 2017b; Wilkinson and Nelson 2021).

In humans, alpha activity, including a dominant peak frequency of approximately 10 to 13 Hz (upper alpha), has specific functional relevance across several major neurophysiological systems in typically developing individuals (Bollimunta et al. 2011; Fries 2015; Garcia-Rill et al. 2016; Jensen and Mazaheri 2010; Zhang et al. 2018). As a radio receiver prefers specific electromagnetic frequencies, neurons, and oscillatory networks also demonstrate frequency preferences (Tseng et al. 2014; Tseng et al. 2006). In several neuropsychiatric disorders, including epilepsy, schizophrenia, neuropathic pain, and tinnitus, a lowering of the dominant peak frequency from the alpha to theta range has been observed (Vanneste et al. 2018). Furthermore, in these conditions, a lower frequency power in the theta range has been more strongly associated with elevations in gamma power. This constellation of findings, a lowering of the dominant frequency toward the theta range with parallel increases in resting gamma activity, has been referred to as the thalamocortical dysrhythmia (TCD, Lakatos et al. 2020; Llinas et al. 2005). In animal models of TCD, hyperpolarization of thalamic relay neurons has been observed to lead to alterations in firing patterns of low-threshold calcium spikes, which are reflected in local field potentials as elevated theta rhythmicity (Jeanmonod et al. 1993; Jeanmonod et al. 2003; Llinás et al. 1999). A dominant theta rhythm may be reflective of a diminished inhibitory capacity at a systems level resulting in excess asynchronous gamma activity and the so-called gamma “edge effect” (Golovin and Broadie 2017; Llinás et al. 1999; Paluszkiewicz et al. 2011; R et al. 2005).

We postulated that in FXS, a slower dominant peak frequency (5 to 7 Hz) might inadequately drive neural ensembles, which prefer typical alpha peak frequencies (8 to 10 Hz), and disrupt the canonical role of alpha in establishing inhibition-timing windows for optimal sensory and neurocognitive processing (Bonnefond and Jensen 2015; Jensen et al. 2014; Jensen and Mazaheri 2010; Zhang et al. 2018).

To study this question at the systems level, we used a data-driven method to profile the spectral properties of resting-state EEG data in a large cohort of individuals with FXS and sex- and age-matched controls. We employed a “bottom-up” approach to classifying coherent spatial and temporal features into empirically derived frequency bands (Cohen 2021). We reasoned if alpha band activity was present in FXS but operated at a lower frequency, we would see the same number of empirically derived frequency bands as controls. Alternatively, EEG patterns in FXS may be more complex, such as merging or adding frequency bands with distinct spatiotemporal features.

**Figure 1:**
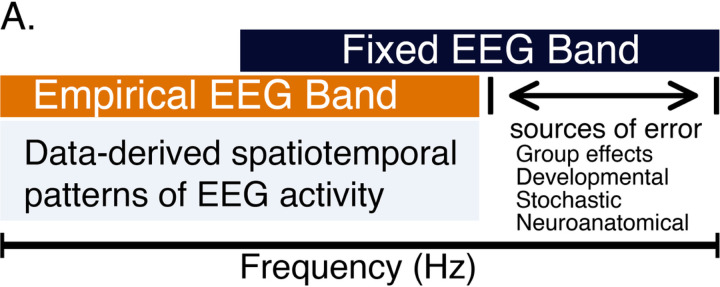
Schematic. A. Utility of data-driven classification of spatiotemporal frequency boundaries in neurodevelopmental disorders in precision medicine applications.

We employed methodology (see Fig. 1) to construct frequency bands derived from spatiotemporal boundaries. Our findings revealed that a widespread frequency shift primarily characterized alpha band alterations in FXS. Notably, although the number of frequency bands was consistent between FXS and control groups, the spatiotemporal properties within these bands were found to be different. These results provide insight into the characteristics of alpha and theta oscillations in FXS, and their clinical relevance, and suggest a larger role for thalamocortical dysfunction.

## Materials and Methods

### Participants

Resting-state EEG data were collected from participants with FXS (n = 65; 37 males, 28 females) and age- and sex-matched typically developing controls (n = 70; 41 males, 29 females). Six other individuals recruited were not included in analyses: two for insufficient valid data epochs (2 FXS), three for excessive line-noise artifact (1 FXS and 2 controls), and one due to intolerance of EEG acquisition (1 FXS).

### Participant consents

Study procedures were approved by the Cincinnati Children’s Hospital Medical Center Institutional Review Board. Participants provided written informed consent (or assent as appropriate) before participation.

### Clinical diagnosis and measures

Southern Blot and polymerase chain reaction confirmed the diagnosis of FXS. Participants with FXS were excluded if they had a history of unstable seizures (any treated seizure within one year) and scheduled use of benzodiazepines. Controls were free of history or treatment of neuropsychiatric illness, as reported via clinical interview. Intellectual function was assessed using the Stanford-Binet Intelligence Scale 5th Ed. (SBS) (Roid 2003). To capture cognitive variability and avoid floor effects, deviation intelligent quotient (IQ) scores were computed (Sansone et al. 2014). Primary caregivers completed assessments for FXS patients, including the Social Communication Questionnaire (SCQ) (Rutter et al. 2003), Anxiety, Depression, and Mood Scale (ADAMS) (Esbensen et al. 2003), and Woodcock-Johnson III Tests of Cognitive Abilities Auditory Attention subscale (McGrew and Woodcock 2006).

### Dataset availability

Data is available to the public as federally mandated at the National Database for Autism Research (NDAR).

### EEG acquisition and processing

Five minutes of spontaneous resting-state EEG data were acquired at a 1000 Hz sampling rate with a 128-channel geodesic electrode net (HydroCel) using an EGI NetAmp 400 amplifier (Magstim, Eugene, OR). As in previous studies, participants were seated comfortably while watching a silent video (standardized across participants) in order to facilitate cooperation with procedures (Wang et al. 2017b). Data analysis was performed using EEGLAB (14.1.2) and custom MATLAB (2018a) scripts (http://github.com/cincibrainlab/vhtp).) Data was digital zero-phase filtered (*eegfiltnew*) with a low-cutoff of 2 Hz and a notch filter from 55 to 65 Hz (with harmonics removed up to Nyquist frequency of the original sampling rate) to remove line noise (Delorme and Makeig 2004). Our objective was to detect neurogenic activity from the gamma band by following best practices to prevent myogenic contamination (Hipp and Siegel 2013). This approach was recently validated to effectively reduce myogenic contamination from approximately 25–98 Hz (Fitzgibbon et al. 2016). The preprocessing assistant performed continuous cleaning by excluding data segments with large amounts of movement artifact and interpolated bad channels (no more than 5% per subject) using spherical spline interpolation implemented in EEGLAB 14. An artifact subspace reconstruction (ASR) approach with default parameters was applied to apply a reconstruction mixing matrix with non-interpolated neighboring channels (Chang et al. 2020). Eye movements, blinks, and cardiac artifacts were removed via independent component analysis on each dataset using an extended INFOMAX algorithm (Lee et al. 1999; Mullen et al. 2015) with PCA rank reduction (further reduced for interpolated channels). A table of group comparisons of key preprocessing variables (removed channels, artifact components, duration of signal) is included in Supplementary Table 1. For each subject, 120 seconds of artifact-free data (60, 2-second epochs) were analyzed.

### Source separation by generalized eigenvalue decomposition

Generalized eigenvalue decomposition (GED) was used to detect empirical spatiotemporal boundaries in the neural activity (Cohen 2021) from continuous EEG data. First, we constructed channel covariance matrices to capture the linear relationships between pairs of channels and their corresponding amplitude time series. GED was performed between sequential finite-impulse bandpass filtered signal (S) matrices between 2 to 80 Hz in 500 logarithmically spaced steps and the broadband signal (R) matrix for each subject. We settled on 500 frequency steps after several rounds of empirical testing with other values, which provided considerable redundancy of the filtered signals, high resolution for the frequency correlation map, and stable identification of clusters. To create each S-matrix, finite-impulse bandpass filter kernels (implemented in EEGLAB 14.1) with a variable passband ranging from 2–5 Hz through the full frequency range.

GED, in this context, is a source separation method that maximizes the separation between two channel covariance matrices while ignoring common features. With GED, spatial patterns of underlying sources can be derived by minimizing volume conduction and spatial autocorrelation in contrast to univariate electrode analysis (e.g., spectral power topography). An empirical frequency range can then be determined by clustering spatial-temporal components produced by GED. This procedure aims to identify frequency bands (e.g., alpha band activity) on an individual participant basis using spatial topography and oscillation frequency simultaneously. This approach can be useful for individualizing frequency bands for each participant rather than assuming canonical bands so that case-control differences in frequency bands can be identified and related to clinical features.

### Generalized Eigenvalue Decomposition

Matrix decomposition by GED maximizes the linear separation between two square matrices while ignoring common features. GED is advantageous in improving signal-to-noise ratio compared to univariate electrode analysis, as volume conduction and spatial autocorrelation enhance the ability of GED to isolate spatial patterns. Thus, GED has been used to generate a sequence of spatiotemporal components that can be subsequently clustered into empiric frequency bounds. The implementation requires the construction of channel covariance matrices that encode the linear relationships between pairs of channels of an amplitude time series. To increase stability, covariance matrices were calculated from 2-second epochs from 60 trials of cleaned data and averaged. A reference channel covariance matrix (R) (from broadband filtered channel-by-time series, **X**_*b*_) and a series of signal (S) matrices from narrow-band filtered channel-by-time data (**X**_*f*_) by multiplying the mean-centered data matrices by their transpose:

(1) $$S=n^{-1}{\text{X}}_{f}{\text{X}}_{f}^{T}\;R=n^{-1}{\text{X}}_{b}{\text{X}}_{b}^{T}$$

Patterns of narrow-band activity within the time series may represent signals of physiological interest and is the first element in the GED computation as the matrix to be “maximized”. As electrode level data is a linear mixture of activity from intracranial sources (and non-neural signals), the GED separation can effectively act a method of blind source separation. The GED solution of each pair of S and R matrices results in a set of eigenvectors, **W**, and eigenvalues (in the diagonal of Λ).


(2) SW=RWΛ

The eigenvector associated with largest eigenvalue is the channel vector, ${\text{w}}_{max}$, that maximizes the Rayleigh quotient (3) and represents the spatial filter that maximally separates the two covariance matrices:

(3) $${\text{w}}_{max}=\underset{\text{w}}{\text{arg}max}\frac{{\text{w}}^{T}\text{Sw}}{{\text{w}}^{T}\text{Rw}}$$

As noted by Cohen, the mechanism of source separation by GED can be more intuitively represented by Eq. 4, which represents the ratio of narrow-band (S) and broadband (R) signals.


(4) $$\text{W}\Lambda =\frac{\text{SW}}{\text{R}}$$

The gedBounds method incorporates two additional optimizations of the channel covariance matrices to improve the quality of decomposition. First, non-representative covariance matrices at the epoch level were cleaned prior to averaging S and R covariance matrices. For each epoch, the z-scored Euclidean distance was calculated for each covariance matrix per epoch and rejected if greater than three standard deviations from the mean. Second, 1% shrinkage regularization was applied to the R matrix which can improve the decomposition of empirical data (Lotte and Guan 2010).

### Correlation Matrices and Clustering

Following GED, a frequency-by-frequency correlation matrix was constructed from each frequency decomposition’s top eigenvector (or component). The eigenvectors represent a spatiotemporal pattern of activity and can be plotted as a scalp topography. It is assumed that activity within consecutive frequencies comprising a spectral band (i.e., alpha) share underlying neural generators whose topological projection would be similar. However, since volume conduction is minimized, the topography interpretation primarily represents the signals that discriminate the narrow-band activity from the broadband signal. This property enables the correlation map to serve as a method to distinguish neighboring narrow-band activity. Squared correlations were used as eigenvectors do not have an inherent sign.

When plotted as a heatmap, the frequency correlation matrix has a block-diagonal appearance. These blocks represent coherent spatiotemporal activity over consecutive frequencies that can be considered an empirically defined frequency band. A non-hierarchical clustering method, density-based spatial clustering (dbscan), was implemented to search and define each cluster to extract these blocks. The method does not require that the identified frequency boundaries remain adjacent and unclustered if an activity is poorly correlated. Though the number of clusters does not have to be predefined, a key parameter for dbscan() is the value of epsilon which is the granularity of the resolution in searching for neighborhoods and can influence the number of identified clusters. We implemented the optimization method used in Cohen (21) to identify an appropriate epsilon by maximizing the following quality function (5):

(5) $$q_{\epsilon }=\frac{r_{w}}{r_{o}}+\log p$$

The quality measure *q*_*ϵ*_ is based on the ratio of the average correlation within the cluster, *r*_*w*_, with average correlation outside the cluster, *r*_*o*_. If the proportion, *p*, of the cluster comprises a larger portion of the entire correlation matrix, then the log*p* function will increase the value of the quantity measure or moderate the effect of very small (but highly correlated) clusters.

### Subject-level clustering of frequency components

For each subject, the results of sequential GED between S and R matrices generated a frequency correlation matrix that represents coherent spatiotemporal activity over consecutive frequencies (Fig. 1A). To generate individual frequency band characterization, a non-hierarchical clustering method, density-based spatial clustering (*dbscan*), was implemented to search and define each cluster. The number of clusters was not predefined. Instead, the value of epsilon, which is the granularity of the resolution in searching for neighborhoods, was optimized for each subject based on a systematic quality function (Cohen 2021). For each cluster, we identified the precise lower and upper empirical frequency boundary. Next, for each subject, we smoothed the lower and upper boundary vectors by applying a kernel density estimator (KDE; 2 Hz full-width half-maximum Gaussian kernel). With this approach, a continuous likelihood estimate of true frequency boundaries was created, allowing for averaging of data across subjects and comparisons between groups.

### Derivation of alpha source components

**Figure 2:**
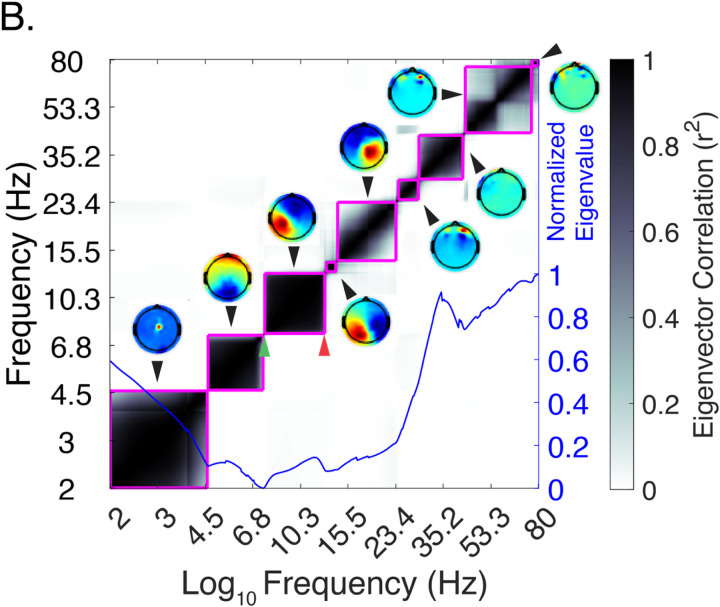
Single subject empirical bounds exemplar. The heat plot depicts the strength of spatiotemporal linear correlation coefficient between narrow frequency filtered and broadband signal channel co-variance matrices (see Methods). Coherent blocks of distinct patterns of neural activity are delineated by a clustering algorithm (magenta line). Each block contains a lower (green arrow) and upper (red arrow) detected boundary. Topographic source projects constructed from PCA-averaged eigenvectors are placed adjacent to heatmap clusters. The magnitude of the separation between narrow and broadband matrices is represented by eigenvalues (blue line).

With a primary focus on alpha-band activity, each participant’s empirical alpha component was defined as the spatial-temporal cluster whose lower boundary is closest but does not exceed 10 Hz and the corresponding upper boundary. Spectral power: Absolute power (µV^2^/Hz) was computed for the derived alpha component time series using Welch’s power spectral density (using MATLAB *pwelch.* Relative power was defined as the proportion of absolute alpha power in the total absolute spectral power of the broadband signal. Topography: To capture the degree of similarity of the spatial topography if empirical alpha components, we calculated the shared spatial correlation between two “ideal” 128-channel topographic templates by applying a Gaussian centered at electrode FCz (“Anterior preference”) and PCz (“Posterior preference”) (see Fig. 2B) (Zuure et al. 2020). As the alpha component has uncertainty in sign, the resulting correlations were squared to obtain shared spatial variance (r^2^).

### Statistical Testing

Statistical analyses were performed in R (version 4.1). Significance was set at p < .05 for all tests and adjusted for multiple comparisons using a 5% false discovery rate(Benjamini and Hochberg 1995). Group comparisons of participant demographics, QC metrics, and component number were compared using a one-way analysis of variance ANOVA. To examine qualitative differences in frequency component boundaries and alpha component topography, multi-level linear mixed effects models (LME) were conducted using the *lme4* package (Bates et al. 2014). In each model, the subject was included as a random effect. Model details are presented adjacent to each result. Post-hoc contrasts of least-square means, with 5% false discovery rate corrected p-values for significance testing, were calculated using the *emmeans* package. Partial correlations were used to examine age-corrected relationships between clinical and neurophysiology features with 5% FDR p-value adjustment.

## Results

### Participants

The final dataset of resting-state EEG data contained 65 participants with FXS (37 males) and 70 healthy controls (41 males) with demographic and clinical characteristics in Table 1. Cases and controls did not differ in age or sex ratio. Participants with FXS had reduced full-scale intelligence quotient (IQ), verbal IQ (VIQ), non-verbal IQ (NVIQ), and increased impairment in social communication and symptoms of anxiety. NVIQ is a general measure of intellectual capacity useful when verbal ability varies among individuals, as in FXS. EEG preprocessing was blinded to group assignment, and no group differences in epoch number, number of interpolated channels, or ICA-derived artifact components were found (see Supplementary Table 1).

### An exemplar of empirical bound detection in a single subject

For each subject, a series of channel covariance matrices were generated from the artifact-free EEG data to succinctly model spatiotemporal relationships for a generalized eigenvalue decomposition (GED). The detection of a series of empirical bands for a single subject is shown in Fig. 2. The heat plot depicts the strength of spatiotemporal correlation (r^2^) between a series of narrow-band frequency channel covariance matrices (S_MATRIX_) and broadband channel covariance matrix (R_MATRIX_). The block-diagonal appearance of the shaded boxes represents the degree of r^2^ of spatiotemporal coherent EEG activity; adjacent boxes demonstrate the distinct spatiotemporal dynamics of distinct activity bands. The magenta line surrounding each shaded box are statistically significant clusters detected by a *dbscan* cluster algorithm. At the bottom of the heatmap, a blue line plot depicts the normalized magnitude of separation (the top eigenvalue per frequency step) between the S_MATRIX_ and R_MATRIX_. Adjacent to each cluster is a topographical plot of principal component analysis (PCA) averaged eigenvectors from each S_MATRIX_ and R_MATRIX_ pair which represents a dipole-like projection of the empirical band of spatiotemporal coherent neural activity. Subject-level derivation plots for all 135 subjects are available in the Supplementary materials.

### Group-level summary of empirical bound detection results

#### Number of detected bands

We first tested if the number of identified empirical EEG bands varied between the groups. The number of detected empirical bands of coherent spatiotemporal activity between 2 and 80 Hz did not differ (mean ± standard deviation) between FXS (M: 12.5 ± 3.4; F: 12.0 ± 3.6) and control (M:12.5 ± 3.8; F: 10.8 ± 3.1; see Table 2). No difference in number of detected bands below 30 Hz between FXS (M: 9.27 ± 2.9; F: 8.8 ± 2.5) and controls (M: 9.5 ± 3; F: 8.5 ± 2.8) was present.

#### Density of detected band boundaries

**Figure 3:**
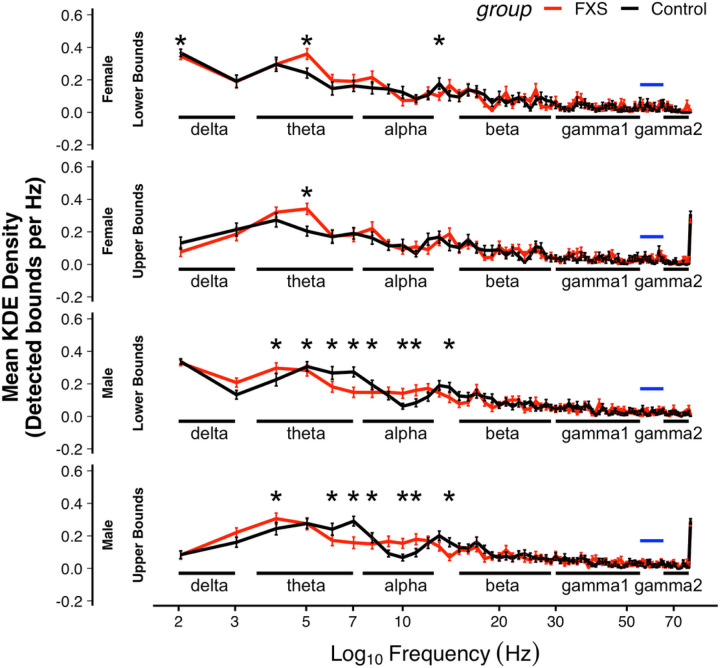
Group comparison. C. Mean kernel density estimates (KDE) of empirically defined frequency boundaries, separated by group and sex. Higher KDE values represent a greater density of detected boundaries per frequency step. Lower and upper bounds were modeled and visualized separately. A three-way interaction effect between Group X Sex X Frequency was present (Lower: F77, 10087=1.72, p=.005; Upper: F77, 10087=1.3, p=.047). Asterisks indicate the statistical difference between the control and FXS groups. Black bars provide a contextual comparison with canonical frequency bands. Blue bars indicate the line noise notch filter.

Next, we estimated the continuous density of the upper and lower frequency boundaries of the detected empirical bands within each group using a kernel density estimator (KDE). A higher KDE value indicated that many participants in the group shared a boundary at a specific frequency. We predicted that the density of frequency boundaries was shifted in FXS and that this difference varies based on sex and by frequency. We ran a linear mixed effect model (LME) in R (version 4.1) using the *nlme* package. We included KDE as the dependent variable and added fixed effects of group, sex, boundary frequency, as well as interaction effects. Subject was included as a random effect. A three-way interaction effect was significant (Lower: F_77,10087_=1.72, p = .005; Upper: F_77,10087_=1.3, p = .047; see Fig. 3). Post-hoc comparisons identified significant sex-matched group differences primarily within the canonical theta and alpha frequency ranges (see Supplementary Table 2). Both males and females with FXS displayed a greater density of boundaries within the theta band (3.5–6 Hz) but differences, as expected, were more pronounced in the male subgroup. In FXS, the peak density for both lower and upper bounds occurred in the lower theta band then remains relatively uniform until an increase in density in the high alpha band (11–12 Hz). As empirical bands in each group shared the same quantity of bands, but displayed shifted frequency boundaries, we next examined individual variation and group differences in spatiotemporal properties.

#### Spatiotemporal uniformity within detected boundaries

The squared correlation coefficient maps (see Fig. 2), which define each detected empirical band represents the uniformity of spatiotemporal activity within a cluster. Overall, no strong correlation between the strength of the spatiotemporal correlation coefficient and the central frequency of each cluster was found (see Supplementary Fig. 1). A weak inverse correlation was found in control females such that higher frequency bands had reduced correlation relative to lower frequency bands (r=-.15, p < .01).

#### Spatiotemporal changes in detected empirical alpha band

**Figure 4:**
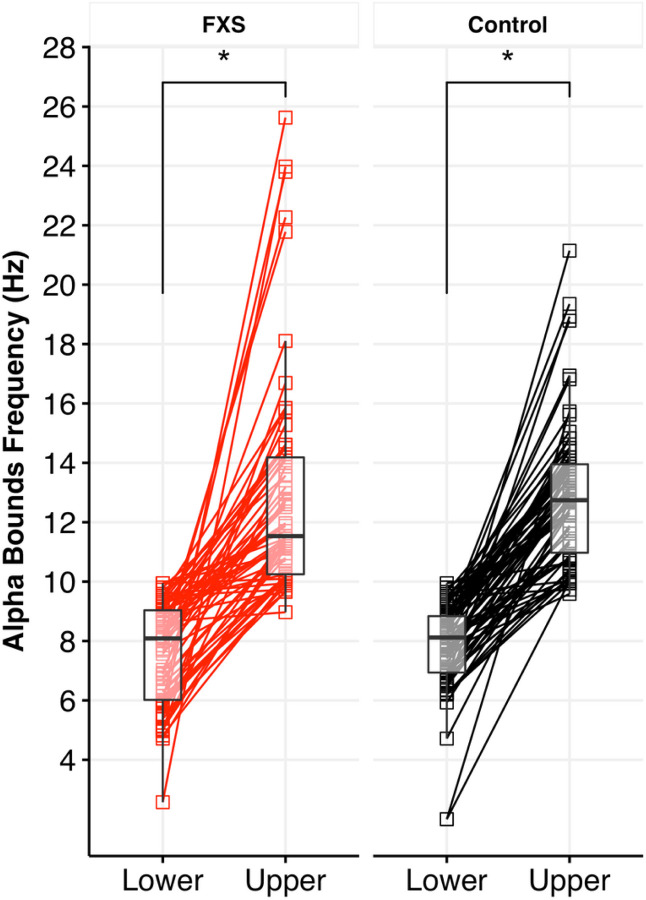
Analysis of empirical alpha component. A. Subject level paired plot of identified lower and upper alpha band boundaries for FXS and control groups. Each alpha band was identified as the closest, but not exceeding, lower bound to 10 Hz. Asterisks indicate the significant within-group difference between the lower and upper bound.

We defined each individual’s empirical alpha band as the detected cluster with the lower frequency bound closest to, but not exceeding, 10 Hz (Cohen 2021). Figure 4 shows the lower and upper boundaries of the individual empirical alpha band by group. The mean detected alpha band ranged in FXS from 7.6 to 13.0 Hz and in the control group from 7.8 to 12.9 Hz. Values in this range correspond approximately to the canonical alpha band. However, in contrast to canonical frequency bands, the empirical alpha band detected by GED involves a temporal (frequency boundaries) and spatial (topography) component.

#### Frequency boundaries of empirical alpha band

We used an LME to examine if detected alpha boundaries varied based on group, sex, or boundary type (upper or lower). The results found a significant main effect of boundary type (F_1,131_=275; p < .001), but no effect of group or sex (see Supplementary Table 3).

#### Extracting empirical alpha band topography

PCA-averaged eigenvector (*w*) of the detected alpha band represents a spatial filter (n-channels in length) that maximally separates alpha range S_MATRIX_ and the broadband R_MATRIX_. When *w* is applied to the alpha range S_MATRIX_ (*w*^T^S_MATRIX_), we can plot the resulting component vector (with length equal to the number of channels) as a scalp topography. Though some of these components may be non-physiological (i.e., noise), certain frequencies will display topographical projections which resemble cortical dipoles of source projections (Haufe et al. 2014).

#### Assessing the distribution of alpha-band topography

**Figure 5:**
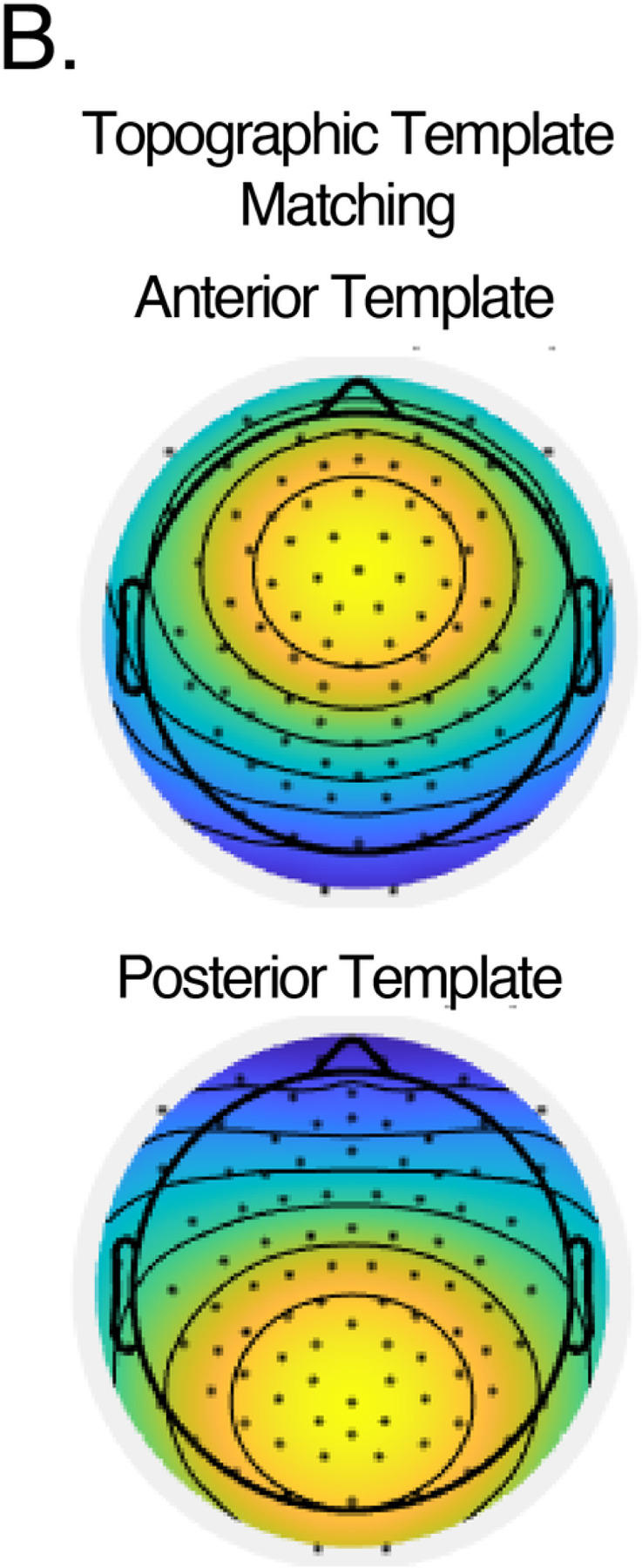
B. Matching templates. EEG templates were created with a Gaussian map centered on electrode FCz or PCz. Templates were used to determine the similarity of neural projection to either anterior or posterior topography.

As each empirical alpha band represents a distinct spatiotemporal configuration, different dipolar projections may represent different anatomical origins (Delorme et al. 2012; Zuure et al. 2020). Specifically, the reduced canonical alpha power and increased theta and gamma power reported in FXS resembles the EEG motif of thalamocortical dysrhythmia seen in certain other neuropsychiatric conditions (De Ridder et al. 2015; Llinas et al. 1999; Vanneste et al. 2018). A defining feature of TCD syndromes is a “slowed” alpha rhythm which has an anterior topography. We predicted that in FXS, the empirical alpha band would also display an anterior topography. We calculated spatial variance (r^2^) to correlate the topography of the empirical alpha band for each subject with either an ideal anterior or posterior template (see Fig. 5) (Zuure et al. 2020). Variance, rather than correlation, is used in this scenario as the eigenvectors produced by GED have uncertainty in sign.

**Figure 6:**
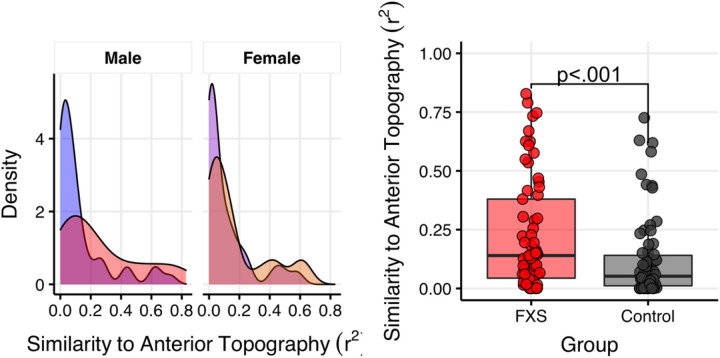
Left. Anterior topography preference for the alpha component in FXS. Density plot of correlations representing subject-level matching of identified alpha component and mid-frontal topology. Right. The topography of the alpha component is significantly altered in FXS participants (F1,131=4.9, p=.028). The alpha component demonstrates a greater degree of similarity to a mid-frontal distribution.

**Figure 7:**
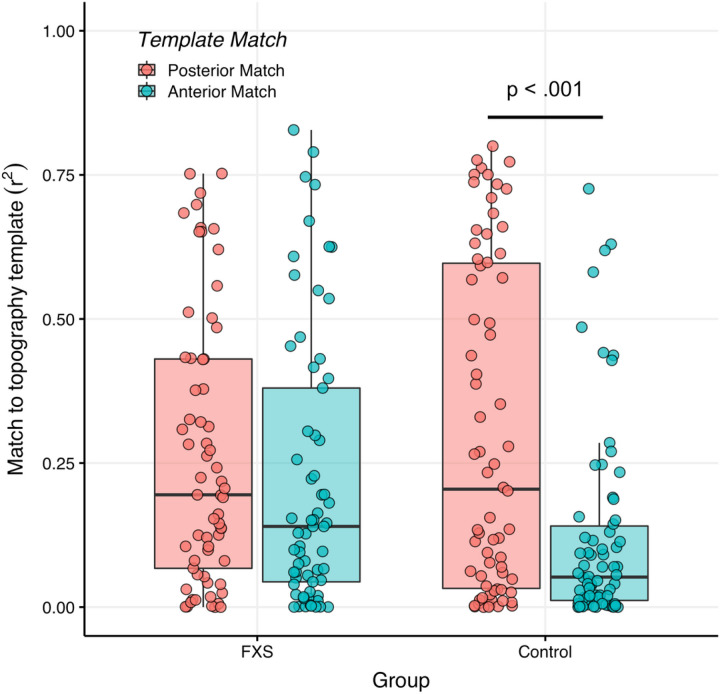
Posterior topography preference for an alpha component in controls. Boxplot depicting similarity of the empirical alpha component to either anterior or posterior topography. A scatter plot (with random horizontal offset for clarity) shows subject-level data. Comparing topography within groups revealed a lack of differentiation among participants with FXS but a posterior topography preference among control participants.

We conducted an LME to examine the effects of group, sex, and template type (anterior or posterior) on r^2^. A two-way interaction effect of group and template type was present (F_1,131_=4.9, p = .028; see Supplementary Tables 4–6). First, between group post-hoc contrasts found that the empirical alpha band in FXS was more closely matched to the anterior template compared to controls (Anterior Template: FXS r^2^-Control r^2^, M = .10, 95%CI [.02,.18], F = 2.36, p = .02, see Fig. 6). Furthermore, within-group contrasts found a significant preference for a posterior template of the empirical alpha component in controls (Anterior r^2^-Posterior r^2^: M=-.18, 95%CI [-.27, − .10], F=-4.19, p < .001), but not FXS participants (Anterior r^2^-Posterior r^2^: M=-.05, 95%CI [-.14, − .04], F=-1.12, p = .26; see Fig. 7.

#### Empirical alpha band power is correlated with auditory attention in FXS

**Figure 8:**
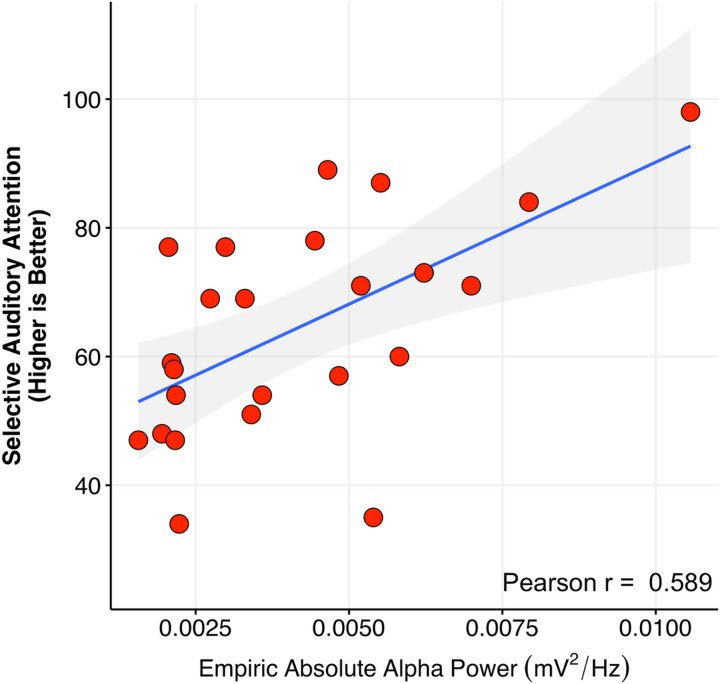
Clinical correlation: Higher performance in auditory attention was positively correlated with increased relative alpha power in males with FXS. Alpha power was empirically derived with subject-level boundaries visualized in Figure 2A. A simple correlation plot is presented for visualization only. Adjusted age-corrected results presented in text (r_partial_=.57, p<.001, n=32, 5% FDR p < .05).

Following the identification of altered topography of alpha component participants affected by FXS, age-adjusted clinical correlations were explored. The male-only subset was considered *a priori* as they exemplified a subset with minimal to no FRMP expression. We performed Pearson’s correlations of intelligence subscales, measures of neuropsychiatric symptoms, and an auditory attention task commonly used in FXS research (see Methods for details) with derived features from their individualized alpha component. In males with FXS, following 5% FDR correction and controlling for age, auditory attention was positively correlated with absolute alpha power (r_partial_=.57, p < .001, n = 32, 5% FDR p < .05, see Fig. 8 for simple correlation plot). No clinical correlations emerged associated with topography. The full table of uncorrected correlations is presented in Supplementary Tables 7 and 8.

## Discussion

Alpha oscillations are among the most prominent and physiologically relevant EEG patterns in the human brain. Marked alterations in alpha oscillations have been reported in FXS, including a leftward shift (lowering) of peak alpha frequency (PAF) and decreased global alpha power (Smith et al. 2021; Van der Molen and Van der Molen 2013; Wang et al. 2017b). It remains unclear, however, whether alpha activity differs between FXS and controls in terms of the frequency range it covers or its spatial configuration. Using a data-driven approach, we used spatiotemporal to classify patterns of neural activity from resting-state EEG into empirically defined frequency bands. We found participants with FXS displayed: 1) similar total quantity of empirical activity bands as controls, 2) a “slower” dominant rhythm with a redistribution of lower-frequency boundaries, 3) an anterior topography of the empirical alpha band, and 4) significant clinical correlation of empirical alpha band activity with neurosensory dysfunction. This study clarifies key aspects of the nature of alpha oscillations in FXS and raises novel hypotheses related to the role of thalamocortical function in causing the heightened neural excitability in FXS, which is largely unknown.

### Incorporating spatial dimension to frequency band classifications

The quantity of detected empirical activity bands did not differ between FXS and control groups. Thus, both groups had similar total divisions of neural activity, though with key differences in the upper and lower frequency limits and the spatial topography of these empirically defined bands. Compared to six to eight canonical frequency bands commonly reported, the quantity of detected empirical frequency bands for each group was approximately twelve. There is a likely reason for this increase: empirical activity bands model different spatial configurations within a frequency range. For example, multiple empirical bands with distinct topographies may fall within the range of a single canonical frequent band. This can be clearly seen in the subject-level tracings (see Figure S2). In both groups, fewer spatiotemporal coherent bands were identified at greater than 30 Hz, in which narrow-band activity may not sufficiently separate from the background signal. Regarding alpha band activity, the mean lower (7.5 Hz) and upper (13 Hz) boundaries of the empirical alpha band in both FXS and control groups were remarkably consistent with the canonical alpha band. The present findings support delineating brain activity by frequency bands, as commonly performed with canonical frequency bands. However, adding a spatial dimension to account for variability in EEG activity may enhance the ability to model heterogeneity and provide insight into physiology. Various forms of data-driven analysis of EEG data have been successfully employed to model heterogeneity in neurodevelopmental populations. For example, across participants with neurodevelopmental conditions, a decrease in individual peak alpha frequency (IAPF) has been reported (Dickinson et al. 2018; Dickinson et al. 2019; Wang et al. 2013; Wang et al. 2017a).

### Altered distribution of empiric alpha frequency boundaries in FXS

Participants with FXS have a higher density of empirical band frequency boundaries in the canonical theta range, and in males with FXS, a sharp reduction of boundaries in the canonical alpha range. This suggests, especially in males with FXS, a leftward shift or “slowing” of the dominant frequency from the alpha to theta frequency range (see Fig. 1C). The narrow-band activity of spectral peaks is thought to reflect the compound synchrony of thousands to millions of neuronal currents. Based on the comparable number of empirical bands between the groups, these results point to a redistribution of neural activity towards lower frequencies.

### Altered configuration of empiric alpha topography in FXS

Spatially, alpha oscillations in FXS had a more anterior projection than in controls. In addition, within the FXS group, the distribution of alpha oscillations in FXS lacked the posterior-to-anterior gradient seen in healthy individuals. This analysis defined empirical alpha bands as the range of coherent spatiotemporal activity around 10 Hz. This approach ensured we captured a frequency typically defined within the canonical alpha band, with the addition of individualized lower and upper boundaries. The isolated empirical alpha components we analyzed represent a source projection rather than scalp alpha activity. GED, when applied to a narrow band and a broadband signal matrix, is a form of linear source separation. The topographical plots, though reminiscent of spectral power plots, more accurately represent areas in which GED minimizes commonalities (e.g., volume conduction) and boosts differences between signals. The final topographies will capture noise or non-neural activity but can also effectively capture dipole components of brain activity. In this sense, these findings may indicate alterations in alpha source generators, which can be observed as spatiotemporal changes in the empirical alpha band.

### Association of empirical alpha activity with sensorineural function

In individuals with FXS, particularly in males, the auditory attention task of the Woodcock-Johnson III was associated with absolute “individualized” alpha power. The auditory attention task does not rely on caregiver reports and measures speech-sound discrimination and resistance to auditory stimuli (McGrew and Woodcock 2006). We speculate that alpha power may serve to index behaviors in which thalamocortical pathways may take a central role, such as in perceptual or attentional tasks (Bollimunta et al. 2011; Foxe and Snyder 2011). No other behavioral correlations survived multiple comparisons and may indicate that higher-order cognition and behavioral reports may not be easily modeled by a single EEG measure.

### Evidence for thalamocortical dysrhythmia in FXS

The slowing and shift of alpha activity from posterior to anterior electrodes have been previously reported with modulation of thalamocortical networks via anesthetic drugs and in pathological states (Vijayan et al. 2013). These features and increases in gamma power associated with cross-frequency coupling (CFC) have been described as a neurophysiological motif called thalamocortical dysrhythmia (TCD). TCD was first described in magnetoencephalography tracings from neuropsychiatric conditions, including depression, epilepsy, neurogenic pain, tinnitus, and Parkinson’s disease (Llinas et al. 1999). Thus far, electrophysiological findings drawn from FXS are consistent with TCD dynamics, including elevated gamma activity (Ethridge et al. 2016; Van der Molen and Van der Molen 2013; Wang et al. 2017b). In essence, evidence from invasive recordings and theoretical models suggest that TCD disorders have alterations in cortical and thalamic synchrony, leading to alterations of thalamocortical drive and regulation of alpha oscillations (De Ridder et al. 2015; Martire et al. 2019; Vanneste et al. 2018).

### Consequences of alpha-band alterations

What significance would spatiotemporal changes in low-frequency activity have on neural function in FXS? Theta and alpha activity transverse the cortex and have been associated with cognitive, emotional, and physiological functions (Zhang et al. 2018). These “traveling waves” are thought to coordinate activity, including binding cell assemblies, multiplex information, and selectively process data (Jensen and Mazaheri 2010; Klimesch 1999; Nunez et al. 2001). For example, in the visual system, the alpha phase can modulate the efficiency of visual information processing, and alpha amplitude is associated with the activity of task-relevant and task-irrelevant regions (Busch and VanRullen 2010). Furthermore, activity within the theta-alpha spectrum is commonly associated with cross-frequency coupling of higher frequency rhythms (i.e., gamma activity) and includes heavy contributions from subcortical sources, especially the thalamus (Canolty and Knight 2010; Lega et al. 2016; Roux et al. 2013). Thus, one reasonable consequence of major alterations in low frequencies may be reduced efficacy of system-level operations that display a frequency preference (Buschman et al. 2012; Canolty and Knight 2010).

### Limitations

The use of GED necessitates specifying parameters that can change the results (Cohen 2021). Despite a systematic approach, varying the search step size (epsilon) can alter band detection. In addition, other methodological decisions, such as filtering approaches and artifact cleaning, may also change the result. To minimize potential selective bias, our preprocessing was blind to the diagnosis group, and we applied the analysis parameters uniformly across groups. Readers can also view each data-driven tracing and access the entire dataset and computer code to reproduce the current results and explore alternative parameters.

## Conclusions

Genetically, individuals with FXS share a common cause but vary greatly in phenotypic manifestations (including sex and mosaic effects). This difference may be due to the contribution of varying gene expression, compensatory processes, or stochastic factors (Baker et al. 2019). FMRP-deficient circuits have significantly altered electrophysiological properties (Goswami et al. 2019; Jonak et al. 2020; Lovelace et al. 2018; Lovelace et al. 2020). Here we used a “bottom-up” approach to empirically model spatiotemporal patterns of brain activity in a large sample of participants with FXS (Cohen 2021). EEG is being used to investigate the links between physiology and behavior and measure target engagement of interventions in NDCs (Ewen et al. 2019; Sahin et al. 2018). Here we provide compelling evidence that even within a monogenetic clinical population, coherent spatiotemporal activity patterns reside within EEG data and contribute to additional variability in group-level analysis. In addition, empirical methods may have increased sensitivity to within-subject designs, such as clinical trials to assess individual responses. Such findings may broaden therapeutic opportunities, including novel neural-targeted “pacemaker” approaches to optimize endogenous rhythms. Such limits impede treatment discovery as drugs leading to the recovery of biological and behavioral functions in Fmr1-/-KO mice have not shown efficacy in clinical trains in humans (Erickson et al. 2018; Harkins et al. 2017).

## Supplementary Material

Supplement 1

## Figures and Tables

**Table 1:** Participant characteristics by group and sex with mean (± standard deviation) group significance testing. FXS, Fragile X Syndrome; y, years; FSIQ, Full Scale IQ; NVIQ, Non-verbal intelligence quotient; VIQ, verbal intelligence scale; Social Score, Social Communication Questionnaire; Anxiety Score, Anxiety, Depression and Mood Scale Anxiety Inventory (ADAMS).

	FXS(F)	FXS(M)	Control(F)	Control(M)	p
	*N=28*	*N=37*	*N=29*	*N=41*	
Age (y)	19.3 (9.48)	22.2 (9.61)	22.2 (12.2)	22.2 (9.81)	0.609
FSIQ	64.7 (28.8)	34.5 (23.2)	99.9 (6.94)	106 (10.1)	<0.001
VIQ	71.4 (26.9)	46.8 (24.6)	97.3 (9.42)	108 (13.3)	<0.001
NVIQ	58.0 (33.2)	22.1 (28.9)	103 (8.65)	104 (12.2)	<0.001
Social Score	9.12 (6.52)	17.4 (6.39)	2.75 (2.57)	1.73 (1.93)	<0.001
Anxiety Score	5.65 (4.68)	7.69 (5.00)	2.41 (3.16)	2.03 (2.22)	<0.001

**Table 2:** Summary of detected frequency boundaries. Group comparison of data-driven frequency boundaries mean (± standard deviation).

	FXS(F)	FXS(M)	Control(F)	Control(M)	p
	*N=28*	*N=37*	*N=29*	*N=41*	
Below 30 Hz	8.79 (2.47)	9.27 (2.91)	8.48 (2.82)	9.51 (3.00)	0.442
Above 30 Hz	3.25 (1.84)	3.19 (1.66)	2.28 (1.33)	2.95 (1.67)	0.091
2–80 Hz	12.0 (3.61)	12.5 (3.43)	10.8 (3.08)	12.5 (3.76)	0.177
