# Supplementary material for: Empirical Frequency Bound Derivation Reveals Prominent Mid-Frontal Alpha Associated with Neurosensory Dysfunction in Fragile X Syndrome
Source: Res Sq. 2023 Apr 28:rs.3.rs-2855646. Preprint. [Version 1] doi: 10.21203/rs.3.rs-2855646/v1 (PMC10168472; doi:10.21203/rs.3.rs-2855646/v1)
Supplement: Supplement 1 [file NIHPPRS2855646V1-supplement-1.pdf]

Supplementary Files

This is a list of supplementary files associated with this preprint. Click to download.

- bpsupplement.docx
